# Evaluation of community level interventions to address social and structural determinants of health: a cluster randomised controlled trial

**DOI:** 10.1186/1471-2458-9-207

**Published:** 2009-06-28

**Authors:** Martin Wall, Richard Hayes, Derek Moore, Mark Petticrew, Angela Clow, Elena Schmidt, Alizon Draper, Karen Lock, Rebecca Lynch, Adrian Renton

**Affiliations:** 1 Institute for Health and Human Development, University of East London, London E15 4LZ, UK; 2 Centre for Social & Health Outcomes Research & Evaluation (SHORE), Massey University, PO Box 6137, Auckland 1010, New Zealand; 3 Infectious Disease Epidemiology Unit, London School of Hygiene & Tropical Medicine, London WC1E 7HT, UK; 4 Institute for Research in Child Development School of Psychology University of East London, London, E15 4LZ, UK; 5 Public & Environmental Health Research Unit, London School of Hygiene and Tropical Medicine, London WC1E 7HT, UK; 6 Department of Psychology, University of Westminster, London W1B 2UW, UK; 7 Centre for Public Health Nutrition, School of Integrated Health, University of Westminster, London W1W 6UW, UK; 8 Department of Public Health and Policy, London School of Hygiene and Tropical Medicine, London WC1E 7HT, UK; 9 Centre for Outcomes Research and Effectiveness (CORE) Research Department of Clinical, Educational & Health Psychology University College London, London WC1E 7HB, UK

## Abstract

**Background:**

In London and the rest of the UK, diseases associated with poor diet, inadequate physical activity and mental illness account for a large proportion of area based health inequality. There is a lack of evidence on interventions promoting healthier behaviours especially in marginalised populations, at a structural or ecological level and utilising a community development approach.

The Well London project financed by the Big Lottery 'Wellbeing' Fund and implemented by a consortium of London based agencies led by the Greater London Authority and the London Health Commission is implementing a set of complex interventions across 20 deprived areas of London. The interventions focus on healthy eating, healthy physical activity and mental health and wellbeing and are designed and executed with community participation complementing existing facilities and services.

**Methods/Design:**

The programme will be evaluated through a cluster randomised controlled trial. Forty areas across London were chosen based on deprivation scores. Areas were characterised by high proportion of Black and Minority Ethnic residents, worklessness, ill-health and poor physical environments. Twenty areas were randomly assigned to the intervention arm of Well London project and twenty 'matched' areas assigned as controls. Measures of physical activity, diet and mental health are collected at start and end of the project and compared to assess impact.

The quantitative element will be complemented by a longitudinal qualitative study elucidating pathways of influence between intervention activities and health outcomes. A related element of the study investigates the health-related aspects of the structural and ecological characteristics of the project areas. The project 'process' will also be evaluated.

**Discussion:**

The size of the project and the fact that the interventions are 'complex' in the sense that firstly, there are a number of interacting components with a wide range of groups and organisational levels targeted by the intervention, and secondly, a degree of flexibility or tailoring of the intervention, makes this trial potentially very useful in providing evidence of the types of activities that can be used to address chronic health problems in communities suffering from multiple deprivation.

**Trial Registration:**

Current Controlled Trials ISRCTN68175121

## Background

The incidence of many chronic diseases such as cancer, cardiovascular diseases, and diabetes is strongly influenced by unhealthy lifestyles including poor diets and sedentariness [1-3]. In the UK, 10% and 3% of all DALYs are attributed to poor diet and insufficient physical activity respectively [4,5]. These translate into direct costs to the NHS of £6 billion for diet and £1.06 billion for physical inactivity related ill-health. These estimates do not include the indirect costs of illness-related lost productivity.

The 2004 Wanless Report 'Securing good health for the whole population'[6] and the 2004 White Paper: 'Choosing Health' [7] emphasised the importance of encouraging individuals to follow healthy lifestyles as a key way to improve health, address health inequalities and to prevent escalation of NHS costs. Healthy eating (HE) and healthy physical activity (HPA) were emphasised as key components of a healthy lifestyle.

There is also increasing recognition that many structural, cultural and environmental factors outside the individual's control constrain their ability to adopt a healthier lifestyle [8]. This is reflected in the development of ecologic models [9] where environmental influences or constraints on behaviour are seen as important determinants of health and health inequalities. Pertinent examples include lack of access to healthy food sources at affordable prices and unsafe environments in which to take physical exercise. Certain ethnic groups also face cultural constraints to their adoption of healthier lifestyles. The recent Sport England survey 'Active People' showed that those who identified themselves as South Asian or African-Caribbean were less likely to meet the recommended levels of physical activity than White European groups [10]. Black Minority Ethnic (BME) communities are often those with the poorest health status.

Low income and poor physical environments also impact on mental health (MH) [11]. Poor mental health is correlated with poor physical health, social exclusion, worklessness and poverty along with other indicators of social or economic stress such as recent migration. Interventions promoting mental health have focused on strengthening the protective factors – a supportive family and a job with some degree of control – and reducing the effect of health risks – such as a family history of psychiatric disorders or financial stress [12]. There is also a literature on the community-level and environmental influences on mental health indicating the potential for interventions promoting wellbeing at these levels[13]. Public spending on mental health services was estimated at £7.9 billion in 2002/3 though again indirect costs will be much higher [14].

However, the evidence for public health interventions aimed at HPA, HE and MH is weak. This is particularly the case for 'complex' interventions, where the 'active component' of the intervention is not clear[15].

Gaps in evidence also exist for programmes aimed at disadvantaged or BME groups, activities to change structural or ecological factors and interventions designed to work through 'community development' approaches. These are the types of interventions implemented under the Well London project presented in this paper.

### The Well London project

The Well London project is a four-year programme that uses a community development approach to deliver a set of complex health interventions aimed at improving HE, HPA and MH in the most deprived neighbourhoods of 20 London Boroughs. The project is led by The Greater London Authority with the London Health Commission and a consortium of partners including Groundwork London, the London Sustainability Exchange (LSx), the Central YMCA, the University of East London, the South London and Maudsley NHS Foundation Trust (SLaM) and the Arts Council for England. These partners together form the Well London Alliance. The project was launched at the end of 2007 and the interventions, developed in detailed consultation with communities, local authorities (LAs), Primary Care Trusts (PCTs) and London strategic bodies, will be delivered in two phases (covering 9 and 11 Boroughs respectively) with a 6 months gap between them. The interventions are largely funded by the Big Lottery 'Wellbeing' Fund, while the evaluation is co-funded by the Big Lottery and the Wellcome Trust.

This paper describes the process and methods used to evaluate the Well London interventions and their impact on HE, HPA and MH in local communities. This paper was written at the end of the interventions design phase.

Interventions delivered by Well London have been drawn from a set of projects suggested by the project partners. The set of possible themed projects included:

*Healthy Spaces*: a project to improve the quality of public space to encourage physical activity and feelings of security and wellbeing within the local area.

*Active Living*: residents are provided with maps informing them of local resources for making healthy choices; including for example farmer's markets and open spaces.

*Be Creative Be Well*: supports and facilitates local cultural activities to foster social networks and social capital.

*Buywell*: a set of interventions to improve access to healthy food choices in the local shops

*Changing Minds*: local people with experience of mental ill-health are recruited to raise awareness of mental health issues and promote understanding of its impact.

*DIY Happiness*: using humour and creativity and based on theories of positive psychology, activities are intended to reduce the impact of stress and increase psychological resources to cope with adversity.

*Eatwell*: a project to improve diet and nutrition by raising awareness of the importance of diet to physical and mental health and making healthy eating easier and more attractive.

The exact mix of activities in each area has been decided upon through priorities identified by residents and complementarity to the facilities and services already provided. In each site Well London has used an identical process to assess needs, develop intervention components and build community capacity and stakeholder commitment to ensure sustainability [16-18]. Each partner organisation leads on projects pertinent to their area of expertise. In addition all communities engage in activities intended to strengthen local capacity and community development initiatives.

Big Lottery resources allow interventions to the annual value of £100,000 per area. A detailed description of the interventions and their components can be found at .

To allow for these interventions and the overall approach to be evaluated, the Well London areas were deliberately selected in a manner which would allow a formal Cluster Randomised Controlled Trial (CRCT). This process (described in more detail in the methods section) involved: a) a decision to work at Lower Super Output area (LSOA) level; b) power calculations to ascertain the number of intervention and control LSOAs required to detect clinically important effect sizes; c) selection of 4 of the most deprived neighbourhoods in each of the 20 target Boroughs (measured using the Index of Multiple Deprivation (IMD)[19]; d) requesting LAs/PCTs to select 2 from the 4 most deprived areas and e) randomising these to generate one intervention and one control neighbourhood/LSOA per Borough.

## Methods/Design

### Study design and population

**Figure 1:**
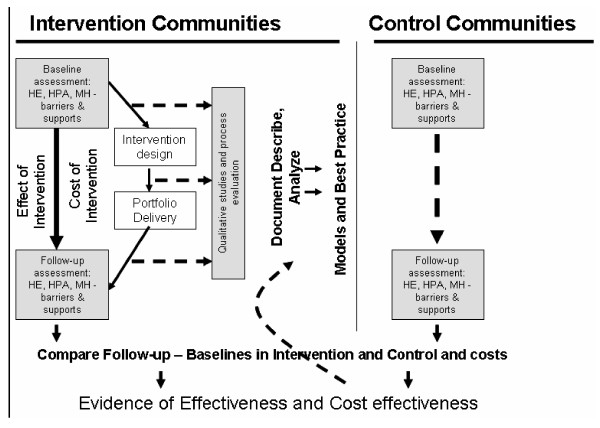
**Evaluation design**.

The study is a cluster randomised controlled trial of an identical community-based approach to needs assessment and design and delivery of complex interventions to promote HPA, HE and MH. There are three components in the evaluation: a) cross-sectional surveys of adults and adolescents using pre and post intervention quantitative measures of HPA, HE and MH; b) a survey of structural and ecological characteristics relevant to health of the intervention areas and c) a complementary qualitative longitudinal study to explore 'how' the interventions affect communities and individuals involved. The implementation of individual projects will also be appraised through a project-specific process evaluation. The overall research design is shown in figure 1.

While the *approach *to the needs assessment, and intervention design and delivery, and the types of intervention delivered will be identical in each experimental cluster, the *exact content *of interventions will be tailored to local needs. Recent literature on evaluation argues that this is valid as "context level adaptation does not mean that the integrity of what is being evaluated across multiple sites is being lost. Integrity defined functionally, rather than compositionally is the key" [20]

Each intervention or control area (cluster) in this CRCT corresponds to a Lower Level Super Output Area (LSOA) a geography created by the UK Office for National Statistics (ONS) following the 2001 Census. Each LSOA contains between 1000 and 1500 residents, has 800 to 1000 residential addresses and covers about 5–6 streets. Information covering social, demographic, economic and health characteristics is available at LSOA level through the ONS' neighbourhood statistics gateway: .

Power calculations were carried out to ascertain the number of matched intervention and control clusters required to detect clinically important effect sizes for indicators of HE, HPA and MH.

The primary outcomes of the interventions in the Well London project are:

- For **healthy eating **a 50% increase in the proportion of adults (base estimate 27%) and a 30% increase in the proportion of children (base estimate 47%) who eat five or more pieces of fruit and vegetables a day.

- For **healthy physical activity**, a 70% increase in the proportion of adults taking 30 minutes of moderate level physical activity 5 times a week (base estimate 18%); and

- For **mental health and wellbeing**, a 30% increase in the proportion of the population achieving key thresholds on mental health and wellbeing indices. The baseline for this index can only be set following the first wave of the adult population survey.

There is limited information available on these primary indicators at the LSOA level. Sample size calculations were therefore based on a number of indirect estimates. The baseline prevalence of HE in adults and children is from synthetic estimates derived from the Health Survey for England by the National Centre for Social Research and are presented at *ward *rather than LSOA level [21]. Using reasonable assumptions about the coefficient of variation between LSOA measures, an 80% power, the effect size given above and formula in Hayes [22] would require 5 pairs of matched clusters.

For mental health the indicator used in the power calculation was the 'per capita rate of claiming of incapacity benefit for reasons to do with mental health', this is available at LSOA level. Using figures derived from the most deprived LSOAs and with an effect size set at half the current claimant rate it was estimated that 9 paired clusters would give the required power. In our study we have 20 paired clusters.

No data on HPA was available at small area level to carry out such a calculation.

Within each cluster a random sample of 100 adults gives sufficient power on both the above measures. Full details of the power calculations are available from the authors on request.

Using the Index of Multiple Deprivation (IMD)[19] all 4765 LSOAs in London were ranked. The most deprived 11% by IMD score were spread across 20 London Boroughs. These Boroughs were then invited to take part in the project and all 20 accepted. In each Borough the four most deprived LSOAs were identified and local authorities and health managers were requested to select two from these four, conditional on the selected LSOAs not bordering each other. One of the two was then randomly allocated to the intervention group and the other to be its matched 'pair' in the control group. Full profiles of the Well London intervention and control LSOAs are at .

The evaluations involve two survey waves; one at the start (2008) and one at the end (2012) of the project. Each wave consists of an adult survey of those above the age of 16 and an adolescent survey of those between the ages of 11 and 16 all of whom live in the selected areas. A qualitative study will investigate and explore in greater depth the effect of the interventions on individuals. This will be undertaken through interviews with a subset of those both participating and not participating in the projects and will take place in 11 of the intervention areas over the course of the project. We will also collect data on the health related quality of the Social and Physical Environment of each area to assess the impact of the structural and ecological aspects of the areas on health outcomes.

### Adult Survey

The baseline data collection period for this survey ran from March 2008 to June 2009. One hundred and fifty non-business addresses were selected from the Post Office Address File (PAF) for each LSOA. Letters were sent to each selected address inviting residents to take part in the survey. Interviewers recruited through local community organisations and job advertising websites were trained prior to the first wave of data collection. They then called on those who had not asked to be excluded. Participants were offered a £10 shopping voucher to compensate them for their time in completing the questionnaire. The target sample size was 100 individuals in each cluster. Non responses were recorded on contact sheets and were broken down into: a) inability to locate address; b) inability to contact eligible adult and c) refusal to participate. No data on individual or household characteristics was collected from refusals.

The survey required informed consent and was approved by the University of East London Research Ethics Committee.

The survey instrument is 28 pages long and is divided into six sections. In the first section standard questions are asked to explore the respondents' socio-demographic and economic characteristics (age, income, education, ethnic group). The second section uses the International Physical Activity Questionnaire [23] to assess self reported HPA through the last seven days. The validity of this measure is known to be high in terms of test-retest and is well correlated with objective measures of HPA. The third section 'wellbeing' measures both negative and positive aspects of psychological mood firstly using the GHQ12 [24,25] and then the 8 item 'Hope Dispositional' scale[26,27]. This scale defines 'hope' as an individual's perceptions of their capacities to conceptualise goals, develop strategies to reach those goals and initiate and sustain the motivation for using those strategies. By looking at 'hope' and 'wellbeing' in a way linked to 'capacity' we connect it to aspects of the environment in which the individual operates and thus make this measure responsive to changes likely to be brought about by the Well London interventions. Section three concludes with some questions exploring social capital (opinions on neighbourhood quality) and social networks. The fourth section asks questions on foodstuffs consumed in the last day. These questions are adapted from the Health Survey for England [28] and their focus is the consumption of fruit and vegetables. Although these questions are insufficient to provide detailed information on nutritional intake at an individual level, data collected in short form questionnaires has been shown to be correlated with consumption recorded in food diaries[29] and is sufficient for analysing group consumption patterns necessary for evaluating the intervention in this trial. Several questions also explore food-related behaviour. The fifth section contains questions on participation in, or attendance at a list of cultural and artistic events drawn from a survey carried out by the Arts Council for England [30].

The final section asks about general health and aspects of health related to quality of life. We use the EQ-5D, a 5-item measure of health-related quality of life developed by Euroquol [31]. This defines health quality across the five dimensions of *mobility*, *self-care*, *usual activities*, *pain or discomfort*, and *anxiety or depression *and using three levels of severity: *no problem*, *moderate *and *severe*. This scale has been tested on a number of patient groups[32,33]. Respondents are then asked about their smoking and drinking habits and their use of health facilities. Finally they are asked to report their own height and weight to allow us to calculate their Body Mass Index (BMI) and their waist measurement for which we provide a tape measure. Self reported BMI has been shown to have reasonable accuracy as an estimate of true BMI [34,35] and waist circumference is the best simple anthropometric measure of total body fat, is better than BMI in that regard [36] and is also the best simple indicator of intra-abdominal fat mass [37-40].

### Adolescent survey

The aim of the adolescent survey is to examine the impact of the initiatives during the critical period of life when children/adolescents develop their health behaviours and habits. We assess levels of psychological and physical health as well as collect data on psychosocial risk factors in adolescents known to be predictive of poor outcomes, including social networks, family factors, peer relations and self esteem.

Because of the ethical considerations raised by fieldworkers interviewing minors in their homes, we use a school-based, self-report approach for this part of the study. Data from the Department of Schools Children and Families' (DSCF) school surveys and census data suggest that on average each LSOA contains 120 school pupils aged 11–16. We aim to recruit sixty resident adolescents in each cluster attending schools in and around the borough. Any school with more than 10 pupils from an LSOA were identified using DCSF annual school survey data and contacted. LSOA resident children may also be attending schools outside their home Borough. Initial estimates based on DCSF data suggest this survey may require the cooperation of up to 150 schools. We attempt to recruit equal numbers of boys and girls with an even age spread from ages 11 to 16 (years 6–11).

We gain appropriate written informed consent. Children then are given a self-administered questionnaire in a classroom situation under the supervision of a researcher and teacher. The questionnaire maps onto the same six domains as the adult survey. However as this survey is a self report survey with an adolescent group who may be as young as 11, we intend only to use measures that have been specifically designed and validated for use with children and adolescents. The survey covers the child's family demographics; aspects of physical and sedentary activity; psychological wellbeing; attitudes to school and the social and physical environment; artistic activities and levels of health and health behaviours.

Demographic questions have been adapted from previous adolescent health surveys [41,42]. Physical activity is assessed with the Physical Activity Questionnaire (Adolescent version) PAQ-A. This is a 7-day recall questionnaire of the extent to which children engage in vigorous physical activities. The PAQ-A has good validity compared to other recall measures[43,44]. We also incorporate questions on health behaviours from the WHO/HBSC surveys of adolescent health across Europe [45].

To measure different aspects of psychological wellbeing and social support we use five standardised scales. The ten item Rosenberg Self-Esteem Scale assesses the well-established relationship between self-esteem and psychological wellbeing (e.g., depression, social anxiety, loneliness, alienation; see Blascovich [46]). Extensive and acceptable reliability (internal consistency and test-retest) and validity (convergent and discriminant) information exists for this scale.

We also use the five item Satisfaction With Life Scale (SWLS) to look at general life satisfaction [47], a construct that is independent of self esteem. Pavot [48] provides an extensive list of studies that have used the SWLS with corresponding normative data. The SWLS has been found to be positively associated with measures of adult subjective wellbeing and negatively associated with measures of psychopathology [47].

We also use the strengths and difficulties questionnaire (SDQ). The SDQ is a valid and reliable measure of adjustment and psychopathology in children and adolescents[49] The SDQ has been used to screen for psychiatric disorders in community samples of children as part of clinical assessment, as a treatment-outcome measure and as a research tool [50-52]. The SDQ correlates highly with teacher/parent reports and with the Child Behaviour Checklist, is sensitive in detecting inattention and hyperactivity and effective in detecting internalising and externalising problems [53].

The survey also employs the PANAS (Positive and Negative Affect Schedule) to assess levels of positive and negative dispositional dimensions. It is predictive of tendencies towards anxiety and depression and is based on the tripartite theory of mental health [54]. Studies have shown the scales to be stable, highly internally consistent and largely uncorrelated [55].

We will use the Multidimensional Scale of Perceived Social Support (MSPSS) [56] to assesses the presence and use of social support networks available to the children. Low levels of social support leaves children at greater risk of negative psychosocial outcomes.

The third section exploring physical and non-family social environment uses questions previously administered in the RELACHs study [41] CHIP-AE and the international WHO surveys of young peoples' health (HBSC -See Currie [45]). The questions focus on social relations with peers and family, experiences of bullying, attitudes to schoolwork, perceptions of threat, thoughts about future prospects and perceptions of the local built environment.

The fourth section requires adolescents to recall what they had to eat over the previous 24 hour period for breakfast, lunch, dinner and for snacks. The section is based on similar studies of food and drink in children[57]. A comprehensive list of possible foods is provided to cue the responses. We ask questions about the context of eating and about general eating and drinking behaviours. We also ask children for their height and weight.

In the fifth section adolescents are asked to recall when they have taken part in creative activities and report on where these took place – at school, home, at a local event or arts centre. Children are also asked to recall the last time they purchased art and attended art related events. These questions are drawn from a survey carried out by the Arts Council for England [30].

The final section will include questions from the RELACHs study[41], the HBSC surveys and from other frequently used health questionnaires. The section will ask in detail about smoking, alcohol and drug use (based on RELACHs measures see Clark[41]) but the main focus is on measuring chronic and stress-related illness.

### Qualitative longitudinal study in 10 intervention areas

The aim of the qualitative study is to elucidate and investigate the causal pathways through which Well London project activities impact on individuals. In particular, it focuses on the effectiveness of the project activities in empowering and enabling people to set goals and to achieve change in relation to the overall project objectives of increasing MH, HE and HPA. As part of this, it also aims to explore the relationships and connections between people and their environment, both physical and social.

The Well London interventions are complex in the MRC sense that the 'active ingredients' are difficult to identify [58]. Springett [59] suggests that such interventions produce a range of outcomes, both intentional and unintentional and the relationship between input and outcome is not clear-cut. Furthermore, the nature of interventions at a community level may evolve as interactions and dynamics in community engagement change during the project. Local environments are themselves 'complex' [60] and certain outcomes may result from mechanisms working in a specific context [61]. Therefore an evaluation should consider not only "what works for whom", but also "in what circumstances". These aspects of evaluating the Well London interventions will not be captured by the CRCT [62] and for this reason a qualitative study of individuals exposed to the Well London interventions has considerable potential to add depth to our understanding of their impact.

Following recommendations by the MRC and others for the evaluation of complex interventions, preliminary conceptual modelling based on empowerment theory [63] will be used to predict how various project components may empower both individuals and communities at different levels and in different domains, and how this will lead in turn to enhanced MH, HPA and healthier food consumption patterns.

The objectives of the qualitative study are to: a) identify the processes whereby project activities impact on individuals; b) map how the different project components affect individuals and enable and empower people to overcome barriers to improving health behaviours; c) identify factors in the social and physical environment which are perceived to act as barriers or supports to adopting healthier lifestyles; d) identify which aspects of the complex interventions are 'active ingredients' and which are not perceived to be relevant and/or effective; e) examine whether some participants are more or less responsive to the interventions and why this is the case; and f) investigate how these influences occur or change over time.

The qualitative study will examine the interaction between changes at the individual level, such as changes in confidence, self-esteem, self-efficacy, trust, stress, sense of control, mental health, life satisfaction and wellbeing, and changes in individuals' behaviour, such as their engagement in community events or involvement in social networks, their patterns of healthy eating and physical activity. These changes will be related to the wider social and physical milieu within which individuals are situated. Participants' experiences and perceptions of the project activities, including the manner in which they are delivered through consultation, perceived active ingredients and barriers will be examined through semi-structured interviews.

The qualitative study will interview individuals in ten of the intervention areas. The interviewees will be purposively selected from those participating in project activities relating to the three key goals of Well London namely HE, HPA and MH. The assumptions behind these themed projects will be mapped as suggested by 'theory of change' approaches [59]. Four to six adults in each area will be interviewed and at baseline will include matched individuals not participating in project activities to provide insight into reasons for non-involvement. As the projects have different target groups, differences by age, gender and ethnicity will be captured. The project participants identified in each area will be followed-up in interviews in years 2 and 3. This will occur whether the individual is still participating in the project or not. In addition, information from project providers regarding the content, intended outcomes and participation in the three project themes within each area will be gathered. Such an approach will allow differences in perception and experience to come through by person, broad project theme and area. This sampling strategy will allow analysis by project, person and place.

Topic guides will be developed for both sets of interviews and are derived from the conceptual framework discussed above. These may be modified between the waves of interviews to reflect emerging issues. Data will be analyzed using thematic content analysis and a longitudinal within-subject analysis to examine changes over time. As well as producing important conclusions in themselves, qualitative findings will later be integrated with results from the quantitative survey instruments and the process evaluation, including the community-level variables and information on variance in intervention delivery. This will allow a more detailed assessment of how mode of delivery and local contexts may modulate the effectiveness of specific components of the intervention activities and will, in turn, inform the evaluation of their replicability and of the likely effectiveness in other contexts.

### Study of Structural and Ecological characteristics

The evaluation takes account of considerable evidence that health and health behaviours are influenced by the social and physical environment (SPE) [8] Interventions to promote health need to act on, and take account of the wider influence of health-promoting and health-endangering characteristics of communities and places. For example, the effectiveness of interventions to promote physical activity is determined not just by how well or how intensively the intervention is implemented, but also by the context – including the physical environment (for example, the availability of green and open spaces) and the social environment (for example, levels of crime; and subjective perceptions of how safe the local area is to walk in). Similarly, the effectiveness of interventions to promote mental health and wellbeing has been shown to be influenced not just by mental health at baseline, but also by a wider range of social determinants such as the quality of urban environments [60]; transport services [61] and social and employment networks, as well as many other characteristics of neighbourhoods.

The Well London activities, will act directly on the SPE characteristics of intervention areas as well as on individual health behaviours and it is therefore necessary to measure changes over the course of the study in SPE at community level as a complement to both the individual-level trial outcome indicators and the qualitative study of individuals interacting within their local contexts. The study will collect and analyse data on the health related quality of SPE, the range of local amenities, facilities and services, community engagement (CE) and social networks at different timepoints before during and after intervention delivery. These data will be collected in four ways.

First we use routine data to construct indicators of health promoting or endangering SPE characteristics of the Well London LSOAs. This data is drawn from databases held by the GLA, The London Health Observatory, LAs and PCTs along with commercial databases, Transport for London and the Metropolitan Police and other sources. Well London already has established relationships with these organizations and has access to much of the data they hold at LSOA level and above. This will allow us to provide initial maps of these indicators in both the control and intervention areas; to characterise the relationship between the environment and health at baseline, and in some cases to assess change in these indicators over time.

Secondly, the delivery of the Well London programme itself provides an important source of qualitative information on community level indicators. The initial CE process has mapped existing amenities and facilities and aspects of SPE which impact health and health behaviour in order to design interventions that complement existing amenities and facilities, fill in gaps in provision and leverage resources to priority areas.

Thirdly, questions on individuals' perceptions of the quality, accessibility and acceptability of the SPE, on the quality of local amenities and services, on CE and social networks are included in the adult survey questionnaire and are an objective of the qualitative longitudinal study.

Finally we will carry out additional primary fieldwork to collect information about the SPE not available through the ways already described. This includes information on the control areas that has not been automatically generated through the Well London design process. A checklist of SPE factors which might influence health will be compiled, together with careful delineation of theoretical causal pathways, using evidence from the literature and CE findings. These will include factors influencing HE (such as differential access to different types of food, distance to shops and exposure to advertising [62,63]), HPA and MH (such as walkability, distance to parks, neighbourhood aesthetics, environmental quality and safety, and access to structured exercise and good quality housing). Checklist SPE factors will be operationalised for measurement by modifying existing tools where these are available (e.g. walkability index), or by developing new tools where necessary. Trained observers will visit intervention and control areas and use a pro-forma to collect data on SPE using a systematic address-based sampling approach before and after intervention delivery. Data will be analyzed to yield a simple index for each SPE factor and changes in these over time will be compared between the intervention and control sites.

### Process evaluation

The process evaluation will assess the implementation and receipt of the interventions and will aid interpretation of the results. In particular it can distinguish between faulty interventions and those badly delivered. It is especially necessary in multisite trials where interventions with the same goals may consist of differing elements and be implemented and received in different ways [64]

The evaluation of the delivery of the Well London programme will be carried out in partnership with the London Health Commission and the Big Lottery Fund and will be in line with commitments made in the Well London Strategy Document [65]. There will be both a continuous process evaluation conducted by the Well London Alliance and external evaluations commissioned in years three and five. The evaluation will take place at two levels, the programme level and the project/LSOA level.

At the **programme **level the goals are to: a) document structures and processes through which the Well London programme is managed, and through which projects are delivered; b) identify critical pathways and steps in these processes; c) identify key challenges and barriers in these structures and processes and document how these were overcome, and d) identify key indicators for the GLA's performance management process. At the **LSOA/project **level the goals are to: a) document and describe key structures and processes involved in the development and management of the Well London programme at LSOA level; b) document and describe key structures and processes involved in the delivery of projects at LSOA level and the mechanisms through which these are anticipated to, and do generate outcomes; c) document outcomes among project beneficiaries; d) identify key challenges and document how these were overcome, and e) inform GLA's performance management, monitoring and review process on key items of data.

Evaluation methods will include: a) analysis of quarterly statistics and information collected on activities delivered relative to plan, on performance and on cost; b) analysis of emerging project documentation to assess coherence with overall Well London objectives; c) interviews with individuals leading and delivering projects to both identify design and delivery processes and to track how projects are anticipated to achieve impact; d) documentation of structures, systems and processes for each project; e) identifying linkages with other projects and critical pathways; f) using focus groups convened as part of the annual community engagement process to explore the extent to which Well London activities are addressing local needs and priorities, and f) multimedia documentation with a particular focus on critical pathways and challenges.

These methods will deliver an overall assessment of the coherence of activity delivered with Well London objectives as well as detailed project specifications and critical path analyses available to inform replication and roll out. They will also provide real-time information on challenges and processes to allow critical reflection and remedial action and materials for marketing future health improvement and health promotion programmes utilising a community development approach.

## Discussion

There is a relative lack of evidence on public health interventions evaluated through randomised controlled trials. One particular challenge with collecting this evidence relates to the difficulty in attributing changes in health to the interventions in question as well as difficulties in dealing with known and unknown confounders. The Well London study has addressed these problems by adopting a cluster randomised controlled trial design. It is thus well-placed to answer questions about the effectiveness of complex community-based interventions on health and wellbeing outcomes. Such CRCTs are also often criticised for taking a "black box" approach to the intervention, and the criticism is sometimes made that this approach is inappropriate for community interventions which need to be tailored to local needs. This CRCT also takes account of such considerations, given the involvement of local communities in the intervention delivery process. The multi-method approach to data collection also allows not just the overall effects to be identified but also the mechanisms through which those changes take place. Moreover the collection of a rich set of data on processes and context will allow a detailed description of the barriers and facilitators to the success or failure of intervention.

The interlinked datasets which will be produced as part of this project will ultimately allow us to assess the conditions which are necessary for the intervention to "work". This will eventually allow us and others to assess the extent to which the findings of the study are generalisable, and to state the limits on its generalisability.

Overall, the large sample size the multidisciplinary multi-method approach and the robust design should help ensure that the study provides valuable evidence of the types of activities that can be used to address chronic health problems in communities suffering from multiple deprivation.

## Competing interests

The authors declare that they have no competing interests.

## Authors' contributions

All authors were involved in conceptualising and designing the research described in this protocol. All authors read and approved the final manuscript

## Pre-publication history

The pre-publication history for this paper can be accessed here:


